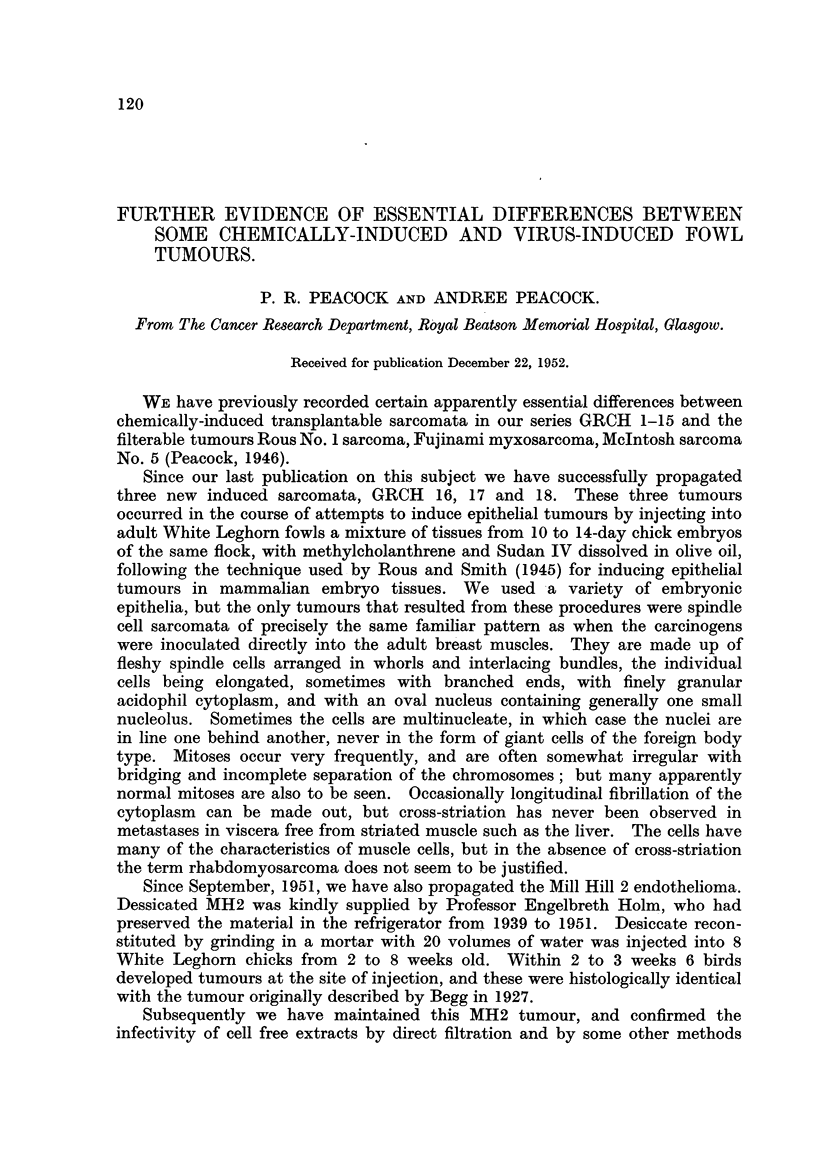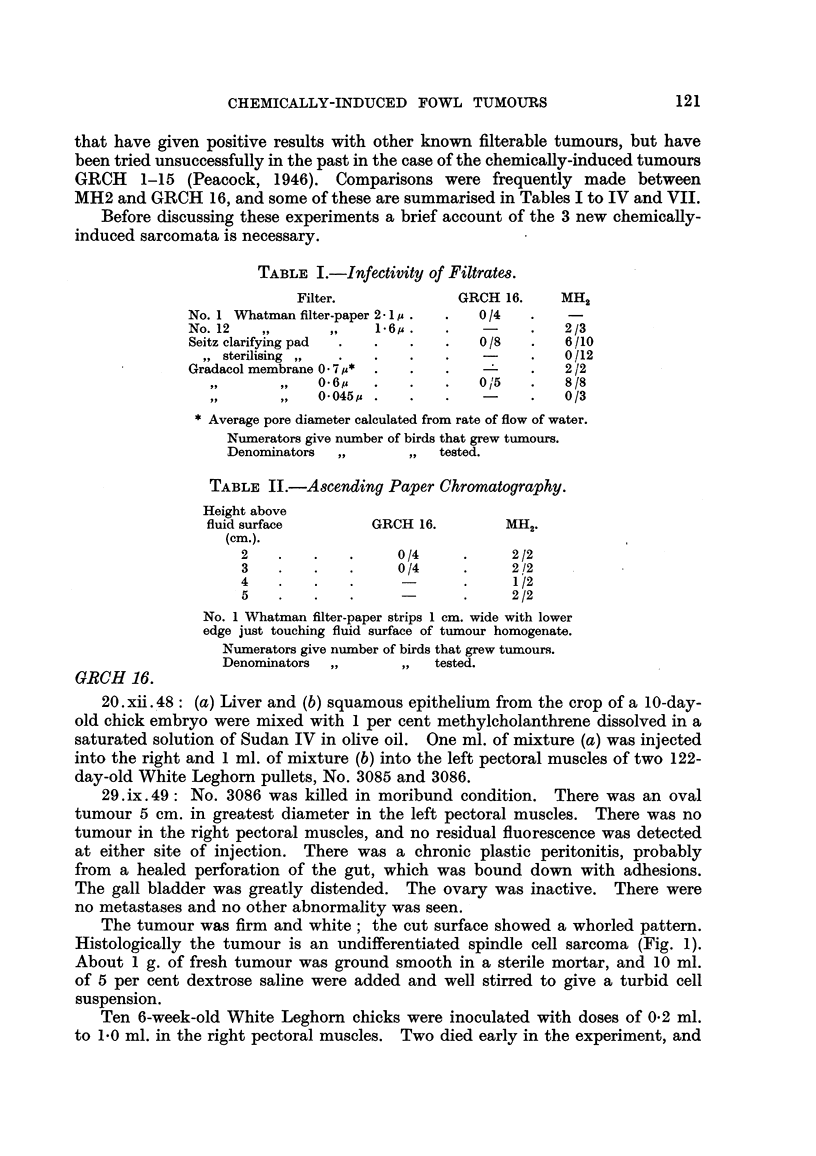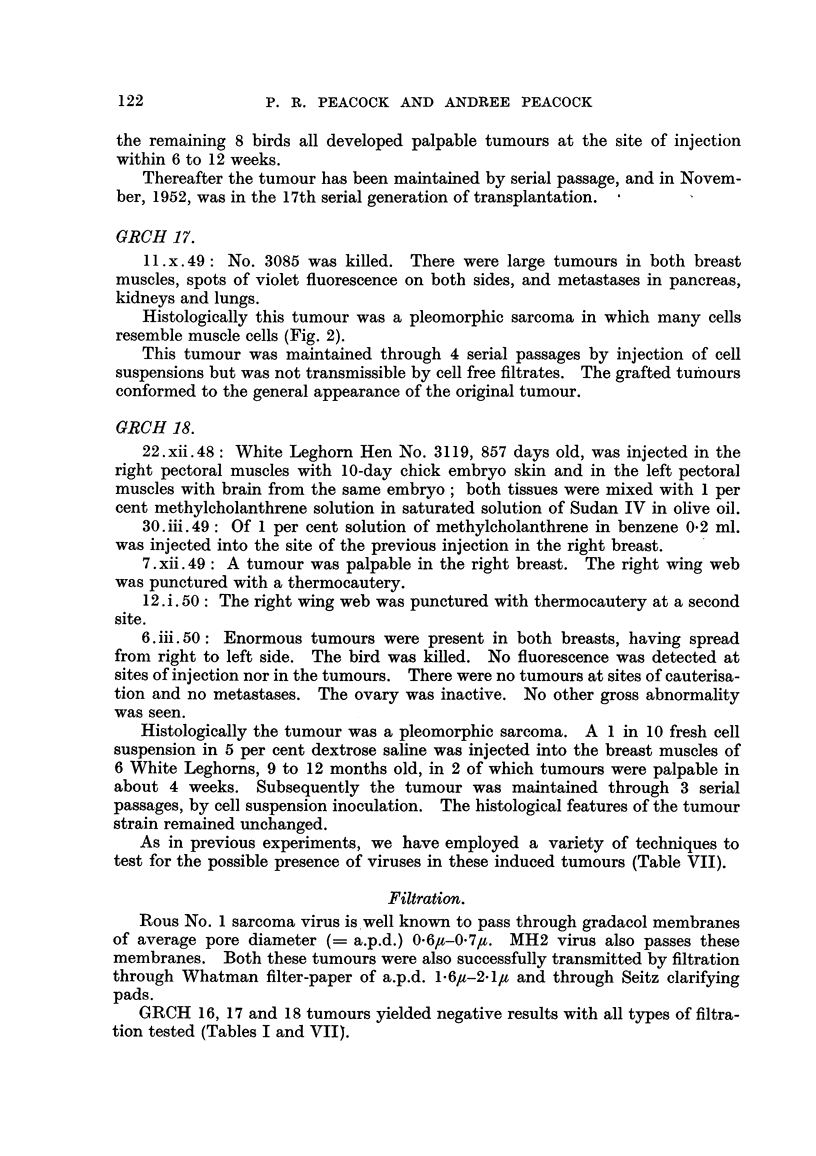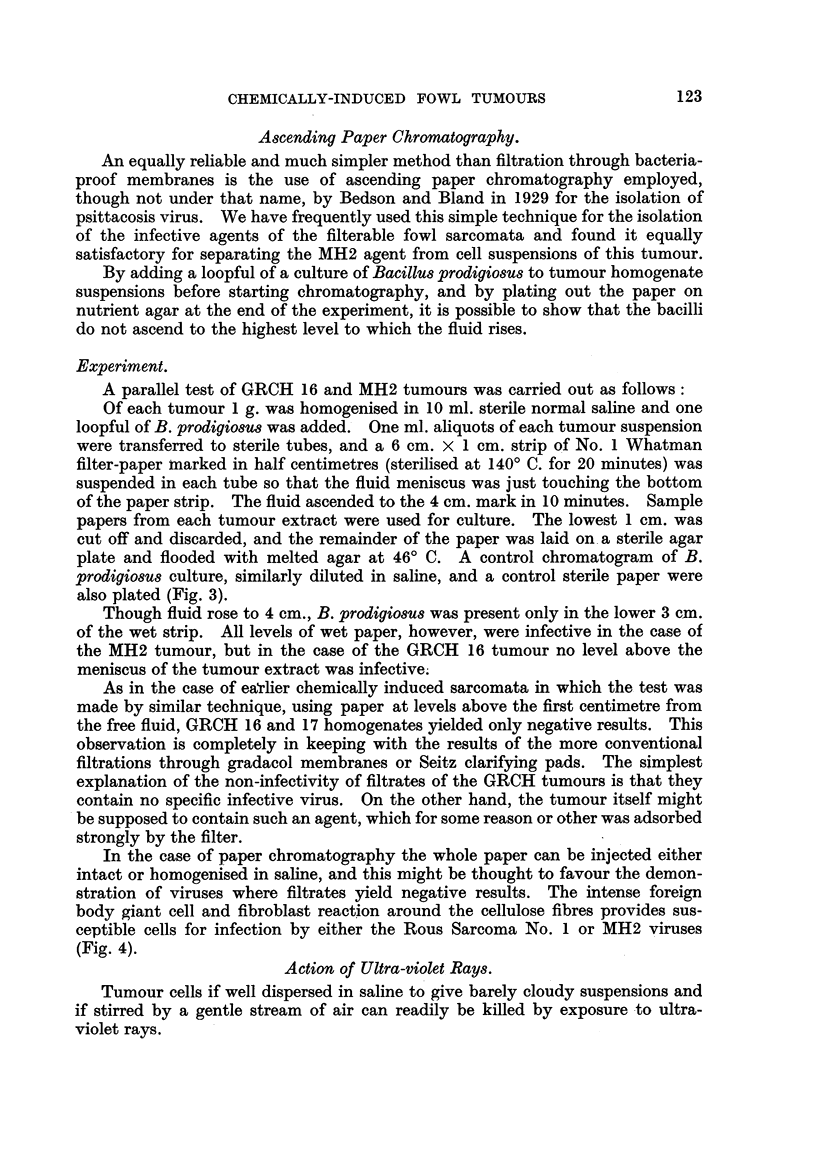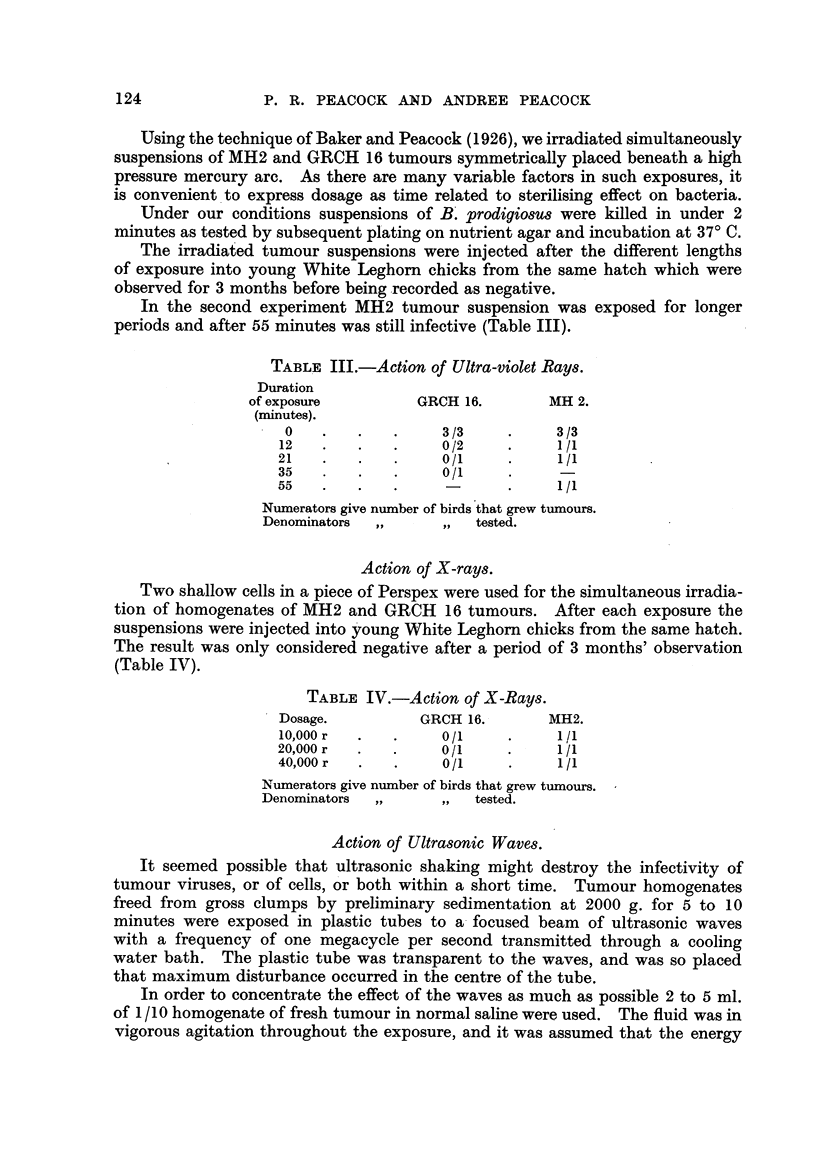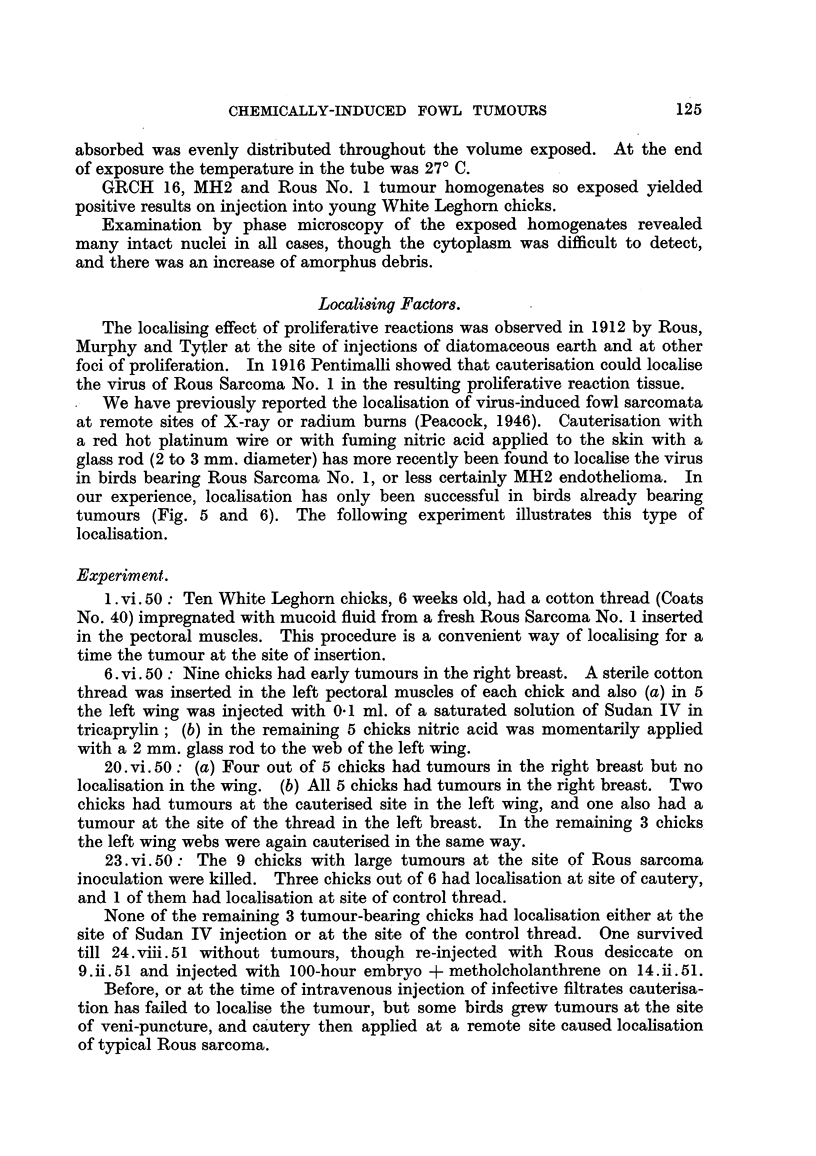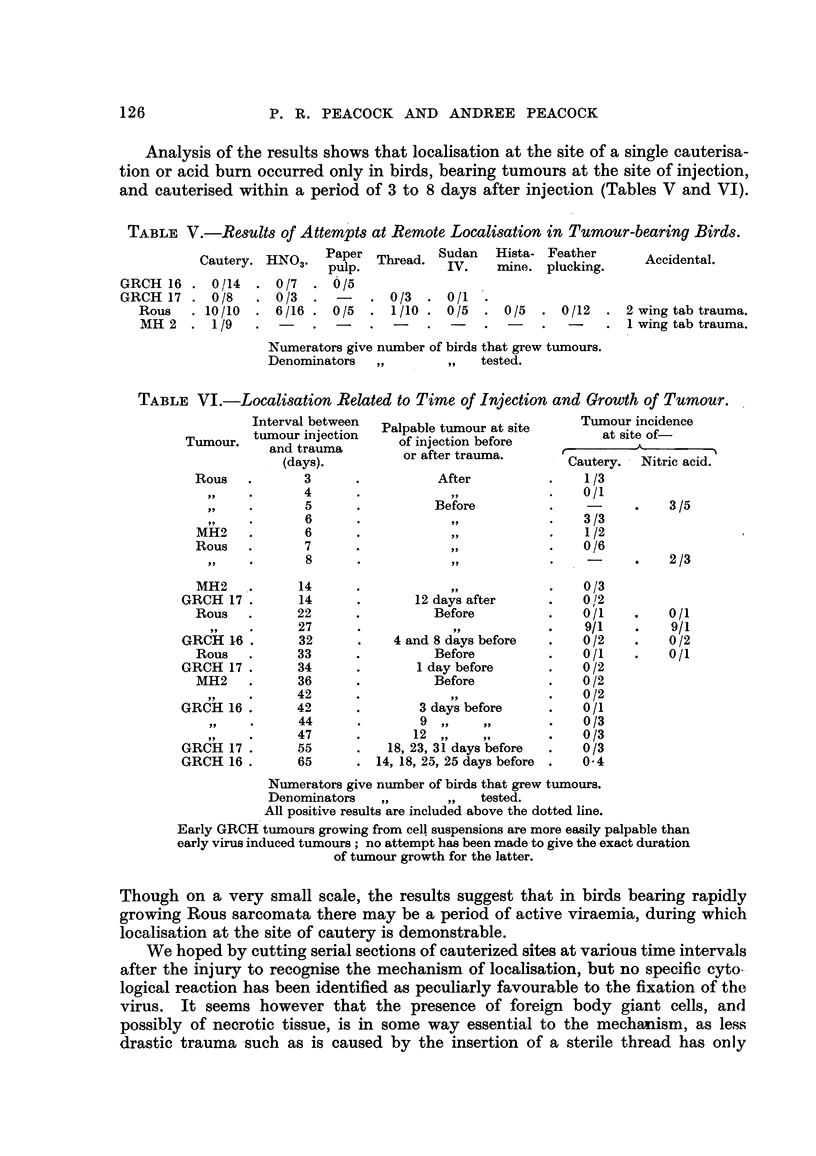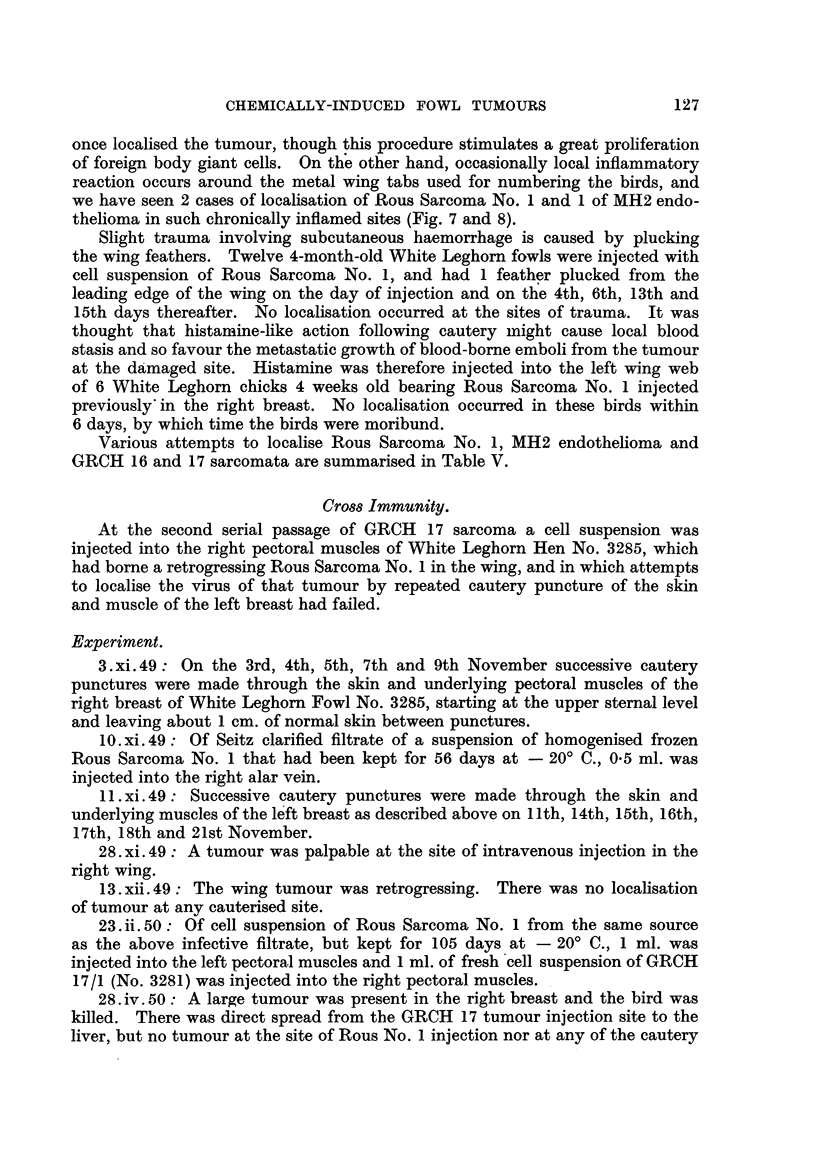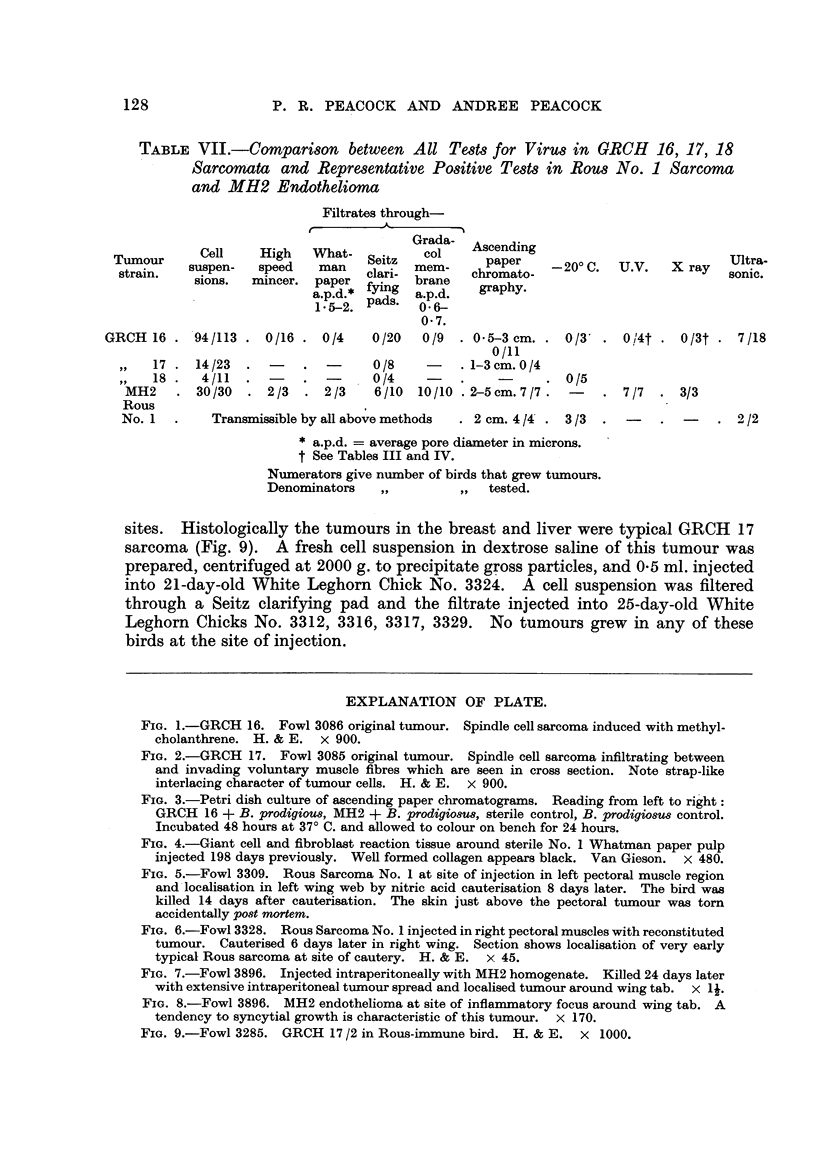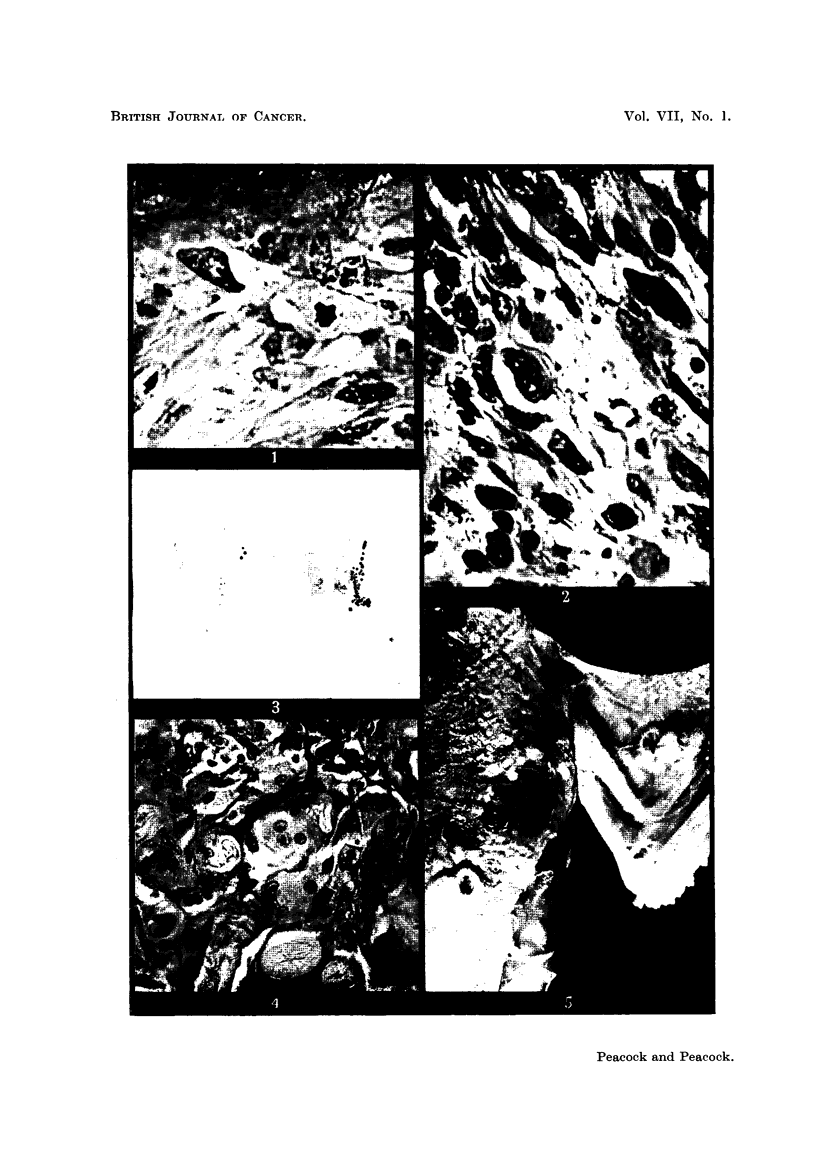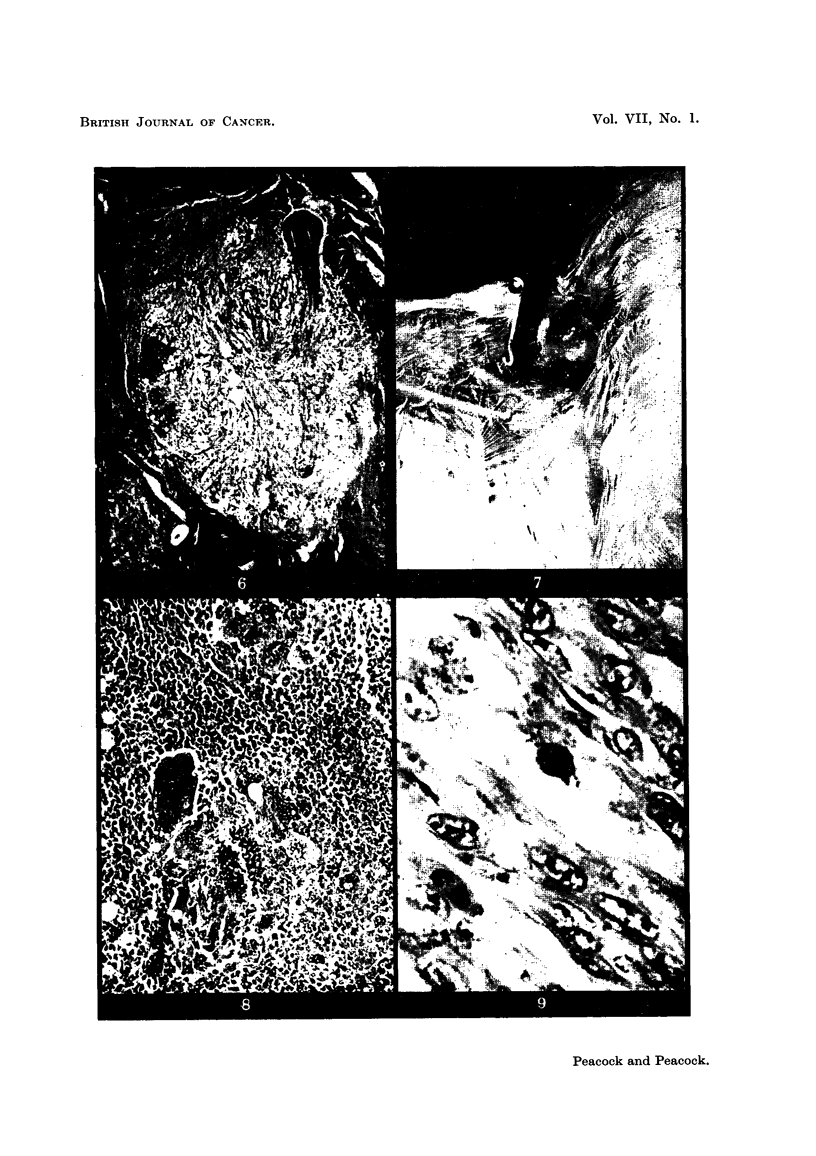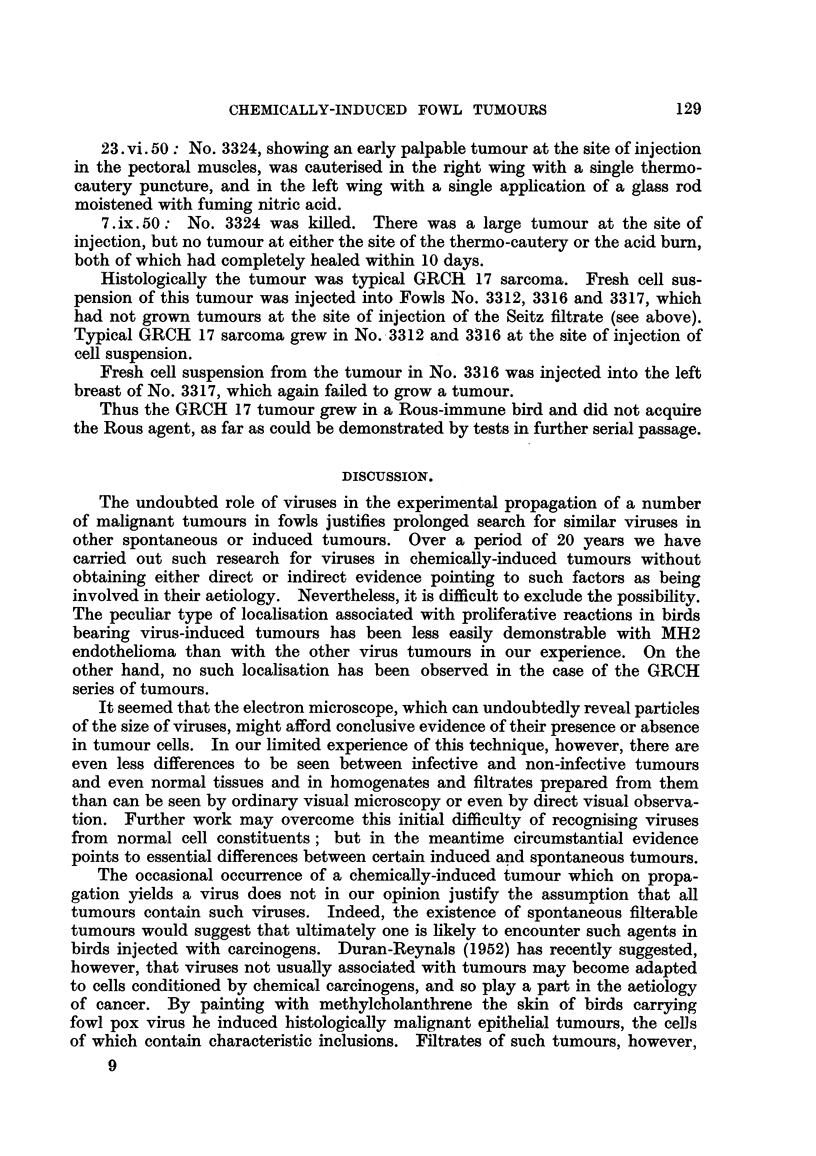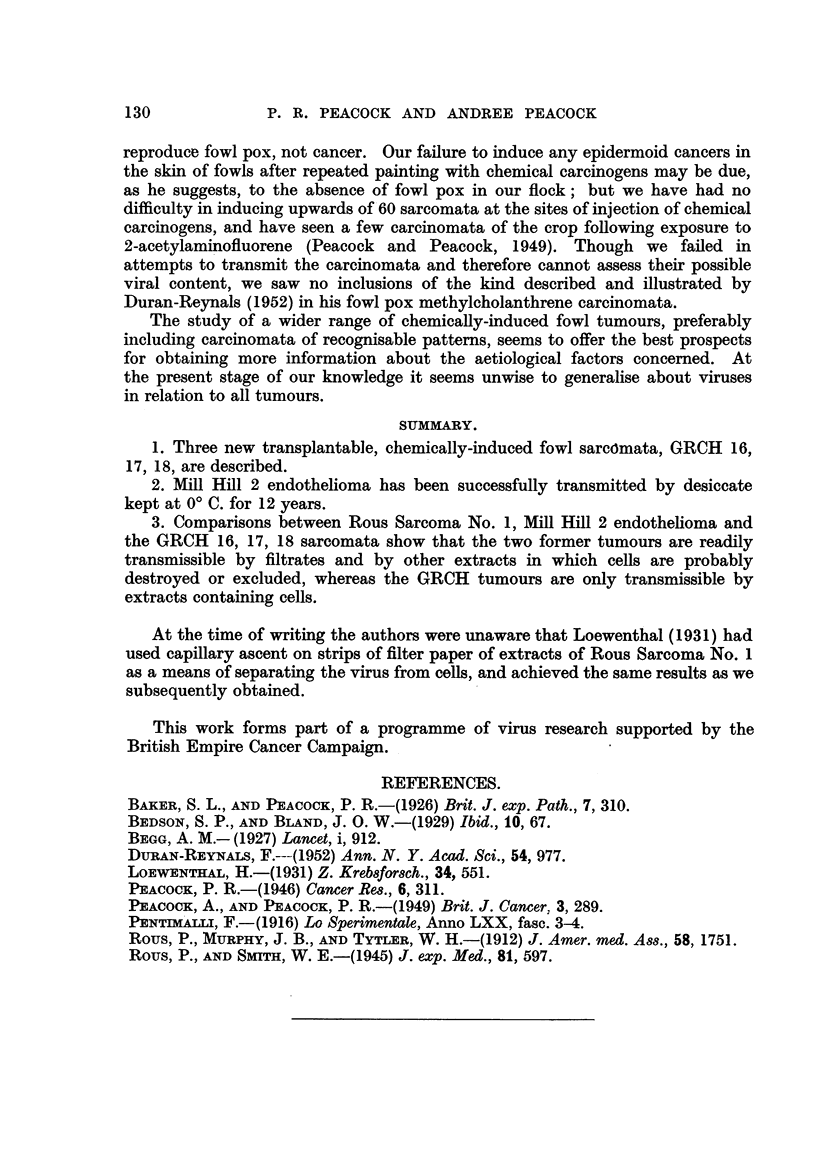# Further Evidence of Essential Differences Between Some Chemically-induced and Virus-induced Fowl Tumours

**DOI:** 10.1038/bjc.1953.10

**Published:** 1953-03

**Authors:** P. R. Peacock, Andree Peacock

## Abstract

**Images:**


### FURTHER EVIDENCE OF ESSENTIAL DIFFERENCES BETWEEN SOME CHEMICALLY-INDUCED AND VIRUS-INDUCED FOWL TUMOURS.

P. R. PEACOCK AND ANDREE PEACOCK.

*From The Cancer Research Department, Royal Beatson Memorial Hospital, Glasgow.*

Received for publication December 22, 1952.

WE have previously recorded certain apparently essential differences between chemically-induced transplantable sarcomata in our series GRCH 1–15 and the filterable tumours Rous No. 1 sarcoma, Fujinami myxosarcoma, McIntosh sarcoma No. 5 (Peacock, 1946).

Since our last publication on this subject we have successfully propagated three new induced sarcomata, GRCH 16, 17 and 18. These three tumours occurred in the course of attempts to induce epithelial tumours by injecting into adult White Leghorn fowls a mixture of tissues from 10 to 14-day chick embryos of the same flock, with methylcholanthrene and Sudan IV dissolved in olive oil, following the technique used by Rous and Smith (1945) for inducing epithelial tumours in mammalian embryo tissues. We used a variety of embryonic epithelia, but the only tumours that resulted from these procedures were spindle cell sarcomata of precisely the same familiar pattern as when the carcinogens were inoculated directly into the adult breast muscles. They are made up of fleshy spindle cells arranged in whorls and interlacing bundles, the individual cells being elongated, sometimes with branched ends, with finely granular acidophil cytoplasm, and with an oval nucleus containing generally one small nucleolus. Sometimes the cells are multinucleate, in which case the nuclei are in line one behind another, never in the form of giant cells of the foreign body type. Mitoses occur very frequently, and are often somewhat irregular with bridging and incomplete separation of the chromosomes ; but many apparently normal mitoses are also to be seen. Occasionally longitudinal fibrillation of the cytoplasm can be made out, but cross-striation has never been observed in metastases in viscera free from striated muscle such as the liver. The cells have many of the characteristics of muscle cells, but in the absence of cross-striation the term rhabdomyosarcoma does not seem to be justified.

Since September, 1951, we have also propagated the Mill Hill 2 endothelioma. Dessicated MH2 was kindly supplied by Professor Engelbreth Holm, who had preserved the material in the refrigerator from 1939 to 1951. Desiccate reconstituted by grinding in a mortar with 20 volumes of water was injected into 8 White Leghorn chicks from 2 to 8 weeks old. Within 2 to 3 weeks 6 birds developed tumours at the site of injection, and these were histologically identical with the tumour originally described by Begg in 1927.

Subsequently we have maintained this MH2 tumour, and confirmed the infectivity of cell free extracts by direct filtration and by some other methods that have given positive results with other known filterable tumours, but have been tried unsuccessfully in the past in the case of the chemically-induced tumours GRCH 1–15 (Peacock, 1946). Comparisons were frequently made between MH2 and GRCH 16, and some of these are summarised in Tables I to IV and VII.

Before discussing these experiments a brief account of the 3 new chemically-induced sarcomata is necessary.

TABLE I.—*Infectivity of Filtrates.*

| Filter. | GRCH 16. | $MH_2$ |
|---|---|---|
| No. 1 Whatman filter-paper 2·1 μ | 0/4 | — |
| No. 12 „ „ 1·6 μ | — | 2/3 |
| Seitz clarifying pad | 0/8 | 6/10 |
| „ sterilising „ | — | 0/12 |
| Gradacol membrane 0·7 μ* | — | 2/2 |
| „ „ 0·6 μ | 0/5 | 8/8 |
| „ „ 0·045 μ | — | 0/3 |

* Average pore diameter calculated from rate of flow of water.

Numerators give number of birds that grew tumours.
Denominators „ „ tested.

TABLE II.—*Ascending Paper Chromatography.*

| Height above fluid surface (cm.). | GRCH 16. | $MH_2$. |
|---|---|---|
| 2 | 0/4 | 2/2 |
| 3 | 0/4 | 2/2 |
| 4 | — | 1/2 |
| 5 | — | 2/2 |

No. 1 Whatman filter-paper strips 1 cm. wide with lower edge just touching fluid surface of tumour homogenate.

Numerators give number of birds that grew tumours.
Denominators „ „ tested.

*GRCH 16.*

20.xii.48 : (*a*) Liver and (*b*) squamous epithelium from the crop of a 10-day-old chick embryo were mixed with 1 per cent methylcholanthrene dissolved in a saturated solution of Sudan IV in olive oil. One ml. of mixture (*a*) was injected into the right and 1 ml. of mixture (*b*) into the left pectoral muscles of two 122-day-old White Leghorn pullets, No. 3085 and 3086.

29.ix.49 : No. 3086 was killed in moribund condition. There was an oval tumour 5 cm. in greatest diameter in the left pectoral muscles. There was no tumour in the right pectoral muscles, and no residual fluorescence was detected at either site of injection. There was a chronic plastic peritonitis, probably from a healed perforation of the gut, which was bound down with adhesions. The gall bladder was greatly distended. The ovary was inactive. There were no metastases and no other abnormality was seen.

The tumour was firm and white ; the cut surface showed a whorled pattern. Histologically the tumour is an undifferentiated spindle cell sarcoma (Fig. 1). About 1 g. of fresh tumour was ground smooth in a sterile mortar, and 10 ml. of 5 per cent dextrose saline were added and well stirred to give a turbid cell suspension.

Ten 6-week-old White Leghorn chicks were inoculated with doses of 0·2 ml. to 1·0 ml. in the right pectoral muscles. Two died early in the experiment, and the remaining 8 birds all developed palpable tumours at the site of injection within 6 to 12 weeks.

Thereafter the tumour has been maintained by serial passage, and in November, 1952, was in the 17th serial generation of transplantation.

*GRCH 17.*

11.x.49 : No. 3085 was killed. There were large tumours in both breast muscles, spots of violet fluorescence on both sides, and metastases in pancreas, kidneys and lungs.

Histologically this tumour was a pleomorphic sarcoma in which many cells resemble muscle cells (Fig. 2).

This tumour was maintained through 4 serial passages by injection of cell suspensions but was not transmissible by cell free filtrates. The grafted tumours conformed to the general appearance of the original tumour.

*GRCH 18.*

22.xii.48 : White Leghorn Hen No. 3119, 857 days old, was injected in the right pectoral muscles with 10-day chick embryo skin and in the left pectoral muscles with brain from the same embryo ; both tissues were mixed with 1 per cent methylcholanthrene solution in saturated solution of Sudan IV in olive oil.

30.iii.49 : Of 1 per cent solution of methylcholanthrene in benzene 0·2 ml. was injected into the site of the previous injection in the right breast.

7.xii.49 : A tumour was palpable in the right breast. The right wing web was punctured with a thermocautery.

12.i.50 : The right wing web was punctured with thermocautery at a second site.

6.iii.50 : Enormous tumours were present in both breasts, having spread from right to left side. The bird was killed. No fluorescence was detected at sites of injection nor in the tumours. There were no tumours at sites of cauterisation and no metastases. The ovary was inactive. No other gross abnormality was seen.

Histologically the tumour was a pleomorphic sarcoma. A 1 in 10 fresh cell suspension in 5 per cent dextrose saline was injected into the breast muscles of 6 White Leghorns, 9 to 12 months old, in 2 of which tumours were palpable in about 4 weeks. Subsequently the tumour was maintained through 3 serial passages, by cell suspension inoculation. The histological features of the tumour strain remained unchanged.

As in previous experiments, we have employed a variety of techniques to test for the possible presence of viruses in these induced tumours (Table VII).

*Filtration.*

Rous No. 1 sarcoma virus is well known to pass through gradacol membranes of average pore diameter (= a.p.d.) 0·6μ–0·7μ. MH2 virus also passes these membranes. Both these tumours were also successfully transmitted by filtration through Whatman filter-paper of a.p.d. 1·6μ–2·1μ and through Seitz clarifying pads.

GRCH 16, 17 and 18 tumours yielded negative results with all types of filtration tested (Tables I and VII).

### *Ascending Paper Chromatography.*

An equally reliable and much simpler method than filtration through bacteria-proof membranes is the use of ascending paper chromatography employed, though not under that name, by Bedson and Bland in 1929 for the isolation of psittacosis virus. We have frequently used this simple technique for the isolation of the infective agents of the filterable fowl sarcomata and found it equally satisfactory for separating the MH2 agent from cell suspensions of this tumour.

By adding a loopful of a culture of *Bacillus prodigiosus* to tumour homogenate suspensions before starting chromatography, and by plating out the paper on nutrient agar at the end of the experiment, it is possible to show that the bacilli do not ascend to the highest level to which the fluid rises.

*Experiment.*

A parallel test of GRCH 16 and MH2 tumours was carried out as follows :

Of each tumour 1 g. was homogenised in 10 ml. sterile normal saline and one loopful of *B. prodigiosus* was added. One ml. aliquots of each tumour suspension were transferred to sterile tubes, and a 6 cm. × 1 cm. strip of No. 1 Whatman filter-paper marked in half centimetres (sterilised at 140° C. for 20 minutes) was suspended in each tube so that the fluid meniscus was just touching the bottom of the paper strip. The fluid ascended to the 4 cm. mark in 10 minutes. Sample papers from each tumour extract were used for culture. The lowest 1 cm. was cut off and discarded, and the remainder of the paper was laid on a sterile agar plate and flooded with melted agar at 46° C. A control chromatogram of *B. prodigiosus* culture, similarly diluted in saline, and a control sterile paper were also plated (Fig. 3).

Though fluid rose to 4 cm., *B. prodigiosus* was present only in the lower 3 cm. of the wet strip. All levels of wet paper, however, were infective in the case of the MH2 tumour, but in the case of the GRCH 16 tumour no level above the meniscus of the tumour extract was infective.

As in the case of earlier chemically induced sarcomata in which the test was made by similar technique, using paper at levels above the first centimetre from the free fluid, GRCH 16 and 17 homogenates yielded only negative results. This observation is completely in keeping with the results of the more conventional filtrations through gradacol membranes or Seitz clarifying pads. The simplest explanation of the non-infectivity of filtrates of the GRCH tumours is that they contain no specific infective virus. On the other hand, the tumour itself might be supposed to contain such an agent, which for some reason or other was adsorbed strongly by the filter.

In the case of paper chromatography the whole paper can be injected either intact or homogenised in saline, and this might be thought to favour the demonstration of viruses where filtrates yield negative results. The intense foreign body giant cell and fibroblast reaction around the cellulose fibres provides susceptible cells for infection by either the Rous Sarcoma No. 1 or MH2 viruses (Fig. 4).

### *Action of Ultra-violet Rays.*

Tumour cells if well dispersed in saline to give barely cloudy suspensions and if stirred by a gentle stream of air can readily be killed by exposure to ultra-violet rays.

Using the technique of Baker and Peacock (1926), we irradiated simultaneously suspensions of MH2 and GRCH 16 tumours symmetrically placed beneath a high pressure mercury arc. As there are many variable factors in such exposures, it is convenient to express dosage as time related to sterilising effect on bacteria. Under our conditions suspensions of *B. prodigiosus* were killed in under 2 minutes as tested by subsequent plating on nutrient agar and incubation at 37° C.

The irradiated tumour suspensions were injected after the different lengths of exposure into young White Leghorn chicks from the same hatch which were observed for 3 months before being recorded as negative.

In the second experiment MH2 tumour suspension was exposed for longer periods and after 55 minutes was still infective (Table III).

TABLE III.—*Action of Ultra-violet Rays.*

| Duration of exposure (minutes). | GRCH 16. | MH 2. |
|---|---|---|
| 0 | 3/3 | 3/3 |
| 12 | 0/2 | 1/1 |
| 21 | 0/1 | 1/1 |
| 35 | 0/1 | — |
| 55 | — | 1/1 |

Numerators give number of birds that grew tumours.
Denominators „ „ tested.

*Action of X-rays.*

Two shallow cells in a piece of Perspex were used for the simultaneous irradiation of homogenates of MH2 and GRCH 16 tumours. After each exposure the suspensions were injected into young White Leghorn chicks from the same hatch. The result was only considered negative after a period of 3 months' observation (Table IV).

TABLE IV.—*Action of X-Rays.*

| Dosage. | GRCH 16. | MH2. |
|---|---|---|
| 10,000 r | 0/1 | 1/1 |
| 20,000 r | 0/1 | 1/1 |
| 40,000 r | 0/1 | 1/1 |

Numerators give number of birds that grew tumours.
Denominators „ „ tested.

*Action of Ultrasonic Waves.*

It seemed possible that ultrasonic shaking might destroy the infectivity of tumour viruses, or of cells, or both within a short time. Tumour homogenates freed from gross clumps by preliminary sedimentation at 2000 g. for 5 to 10 minutes were exposed in plastic tubes to a focused beam of ultrasonic waves with a frequency of one megacycle per second transmitted through a cooling water bath. The plastic tube was transparent to the waves, and was so placed that maximum disturbance occurred in the centre of the tube.

In order to concentrate the effect of the waves as much as possible 2 to 5 ml. of 1/10 homogenate of fresh tumour in normal saline were used. The fluid was in vigorous agitation throughout the exposure, and it was assumed that the energy absorbed was evenly distributed throughout the volume exposed. At the end of exposure the temperature in the tube was 27° C.

GRCH 16, MH2 and Rous No. 1 tumour homogenates so exposed yielded positive results on injection into young White Leghorn chicks.

Examination by phase microscopy of the exposed homogenates revealed many intact nuclei in all cases, though the cytoplasm was difficult to detect, and there was an increase of amorphus debris.

### *Localising Factors.*

The localising effect of proliferative reactions was observed in 1912 by Rous, Murphy and Tytler at the site of injections of diatomaceous earth and at other foci of proliferation. In 1916 Pentimalli showed that cauterisation could localise the virus of Rous Sarcoma No. 1 in the resulting proliferative reaction tissue.

We have previously reported the localisation of virus-induced fowl sarcomata at remote sites of X-ray or radium burns (Peacock, 1946). Cauterisation with a red hot platinum wire or with fuming nitric acid applied to the skin with a glass rod (2 to 3 mm. diameter) has more recently been found to localise the virus in birds bearing Rous Sarcoma No. 1, or less certainly MH2 endothelioma. In our experience, localisation has only been successful in birds already bearing tumours (Fig. 5 and 6). The following experiment illustrates this type of localisation.

*Experiment.*

1.vi.50*:* Ten White Leghorn chicks, 6 weeks old, had a cotton thread (Coats No. 40) impregnated with mucoid fluid from a fresh Rous Sarcoma No. 1 inserted in the pectoral muscles. This procedure is a convenient way of localising for a time the tumour at the site of insertion.

6.vi.50*:* Nine chicks had early tumours in the right breast. A sterile cotton thread was inserted in the left pectoral muscles of each chick and also (*a*) in 5 the left wing was injected with 0·1 ml. of a saturated solution of Sudan IV in tricaprylin ; (*b*) in the remaining 5 chicks nitric acid was momentarily applied with a 2 mm. glass rod to the web of the left wing.

20.vi.50*:* (*a*) Four out of 5 chicks had tumours in the right breast but no localisation in the wing. (*b*) All 5 chicks had tumours in the right breast. Two chicks had tumours at the cauterised site in the left wing, and one also had a tumour at the site of the thread in the left breast. In the remaining 3 chicks the left wing webs were again cauterised in the same way.

23.vi.50*:* The 9 chicks with large tumours at the site of Rous sarcoma inoculation were killed. Three chicks out of 6 had localisation at site of cautery, and 1 of them had localisation at site of control thread.

None of the remaining 3 tumour-bearing chicks had localisation either at the site of Sudan IV injection or at the site of the control thread. One survived till 24.viii.51 without tumours, though re-injected with Rous desiccate on 9.ii.51 and injected with 100-hour embryo + metholcholanthrene on 14.ii.51.

Before, or at the time of intravenous injection of infective filtrates cauterisation has failed to localise the tumour, but some birds grew tumours at the site of veni-puncture, and cautery then applied at a remote site caused localisation of typical Rous sarcoma.

Analysis of the results shows that localisation at the site of a single cauterisation or acid burn occurred only in birds, bearing tumours at the site of injection, and cauterised within a period of 3 to 8 days after injection (Tables V and VI).

TABLE V.—*Results of Attempts at Remote Localisation in Tumour-bearing Birds.*

| | Cautery. | $HNO_3$. | Paper pulp. | Thread. | Sudan IV. | Histamine. | Feather plucking. | Accidental. |
|---|---|---|---|---|---|---|---|---|
| GRCH 16 | 0/14 | 0/7 | 0/5 | | | | | |
| GRCH 17 | 0/8 | 0/3 | — | 0/3 | 0/1 | | | |
| Rous | 10/10 | 6/16 | 0/5 | 1/10 | 0/5 | 0/5 | 0/12 | 2 wing tab trauma. |
| MH 2 | 1/9 | — | — | — | — | — | — | 1 wing tab trauma. |

Numerators give number of birds that grew tumours.
Denominators ,, ,, tested.

TABLE VI.—*Localisation Related to Time of Injection and Growth of Tumour.*

| Tumour. | Interval between tumour injection and trauma (days). | Palpable tumour at site of injection before or after trauma. | Tumour incidence at site of— Cautery. | Nitric acid. |
|---|---|---|---|---|
| Rous | 3 | After | 1/3 | |
| ,, | 4 | ,, | 0/1 | |
| ,, | 5 | Before | — | 3/5 |
| ,, | 6 | ,, | 3/3 | |
| MH2 | 6 | ,, | 1/2 | |
| Rous | 7 | ,, | 0/6 | |
| ,, | 8 | ,, | — | 2/3 |
| MH2 | 14 | ,, | 0/3 | |
| GRCH 17 | 14 | 12 days after | 0/2 | |
| Rous | 22 | Before | 0/1 | 0/1 |
| ,, | 27 | ,, | 9/1 | 9/1 |
| GRCH 16 | 32 | 4 and 8 days before | 0/2 | 0/2 |
| Rous | 33 | Before | 0/1 | 0/1 |
| GRCH 17 | 34 | 1 day before | 0/2 | |
| MH2 | 36 | Before | 0/2 | |
| ,, | 42 | ,, | 0/2 | |
| GRCH 16 | 42 | 3 days before | 0/1 | |
| ,, | 44 | 9 ,, ,, | 0/3 | |
| ,, | 47 | 12 ,, ,, | 0/3 | |
| GRCH 17 | 55 | 18, 23, 31 days before | 0/3 | |
| GRCH 16 | 65 | 14, 18, 25, 25 days before | 0·4 | |

Numerators give number of birds that grew tumours.
Denominators ,, ,, tested.
All positive results are included above the dotted line.

Early GRCH tumours growing from cell suspensions are more easily palpable than early virus induced tumours ; no attempt has been made to give the exact duration of tumour growth for the latter.

Though on a very small scale, the results suggest that in birds bearing rapidly growing Rous sarcomata there may be a period of active viraemia, during which localisation at the site of cautery is demonstrable.

We hoped by cutting serial sections of cauterized sites at various time intervals after the injury to recognise the mechanism of localisation, but no specific cytological reaction has been identified as peculiarly favourable to the fixation of the virus. It seems however that the presence of foreign body giant cells, and possibly of necrotic tissue, is in some way essential to the mechanism, as less drastic trauma such as is caused by the insertion of a sterile thread has only once localised the tumour, though this procedure stimulates a great proliferation of foreign body giant cells. On the other hand, occasionally local inflammatory reaction occurs around the metal wing tabs used for numbering the birds, and we have seen 2 cases of localisation of Rous Sarcoma No. 1 and 1 of MH2 endothelioma in such chronically inflamed sites (Fig. 7 and 8).

Slight trauma involving subcutaneous haemorrhage is caused by plucking the wing feathers. Twelve 4-month-old White Leghorn fowls were injected with cell suspension of Rous Sarcoma No. 1, and had 1 feather plucked from the leading edge of the wing on the day of injection and on the 4th, 6th, 13th and 15th days thereafter. No localisation occurred at the sites of trauma. It was thought that histamine-like action following cautery might cause local blood stasis and so favour the metastatic growth of blood-borne emboli from the tumour at the damaged site. Histamine was therefore injected into the left wing web of 6 White Leghorn chicks 4 weeks old bearing Rous Sarcoma No. 1 injected previously in the right breast. No localisation occurred in these birds within 6 days, by which time the birds were moribund.

Various attempts to localise Rous Sarcoma No. 1, MH2 endothelioma and GRCH 16 and 17 sarcomata are summarised in Table V.

### *Cross Immunity.*

At the second serial passage of GRCH 17 sarcoma a cell suspension was injected into the right pectoral muscles of White Leghorn Hen No. 3285, which had borne a retrogressing Rous Sarcoma No. 1 in the wing, and in which attempts to localise the virus of that tumour by repeated cautery puncture of the skin and muscle of the left breast had failed.

*Experiment.*

3.xi.49*:* On the 3rd, 4th, 5th, 7th and 9th November successive cautery punctures were made through the skin and underlying pectoral muscles of the right breast of White Leghorn Fowl No. 3285, starting at the upper sternal level and leaving about 1 cm. of normal skin between punctures.

10.xi.49*:* Of Seitz clarified filtrate of a suspension of homogenised frozen Rous Sarcoma No. 1 that had been kept for 56 days at — 20° C., 0·5 ml. was injected into the right alar vein.

11.xi.49*:* Successive cautery punctures were made through the skin and underlying muscles of the left breast as described above on 11th, 14th, 15th, 16th, 17th, 18th and 21st November.

28.xi.49*:* A tumour was palpable at the site of intravenous injection in the right wing.

13.xii.49*:* The wing tumour was retrogressing. There was no localisation of tumour at any cauterised site.

23.ii.50*:* Of cell suspension of Rous Sarcoma No. 1 from the same source as the above infective filtrate, but kept for 105 days at — 20° C., 1 ml. was injected into the left pectoral muscles and 1 ml. of fresh cell suspension of GRCH 17/1 (No. 3281) was injected into the right pectoral muscles.

28.iv.50*:* A large tumour was present in the right breast and the bird was killed. There was direct spread from the GRCH 17 tumour injection site to the liver, but no tumour at the site of Rous No. 1 injection nor at any of the cautery

TABLE VII.—*Comparison between All Tests for Virus in GRCH 16, 17, 18 Sarcomata and Representative Positive Tests in Rous No. 1 Sarcoma and MH2 Endothelioma*

| Tumour strain. | Cell suspensions. | High speed mincer. | Filtrates through— What-man paper a.p.d.* 1·5–2. | Filtrates through— Seitz clarifying pads. | Filtrates through— Gradacol membrane a.p.d. 0·6–0·7. | Ascending paper chromatography. | −20° C. | U.V. | X ray | Ultrasonic. |
|---|---|---|---|---|---|---|---|---|---|---|
| GRCH 16 | 94/113 | 0/16 | 0/4 | 0/20 | 0/9 | 0·5–3 cm. 0/11 | 0/3 | 0/4† | 0/3† | 7/18 |
| „ 17 | 14/23 | — | — | 0/8 | — | 1–3 cm. 0/4 | | | | |
| „ 18 | 4/11 | — | — | 0/4 | — | — | 0/5 | | | |
| MH2 | 30/30 | 2/3 | 2/3 | 6/10 | 10/10 | 2–5 cm. 7/7 | — | 7/7 | 3/3 | |
| Rous No. 1 | Transmissible by all above methods | | | | | 2 cm. 4/4 | 3/3 | — | — | 2/2 |

* a.p.d. = average pore diameter in microns.
† See Tables III and IV.

Numerators give number of birds that grew tumours.
Denominators „ „ tested.

sites. Histologically the tumours in the breast and liver were typical GRCH 17 sarcoma (Fig. 9). A fresh cell suspension in dextrose saline of this tumour was prepared, centrifuged at 2000 g. to precipitate gross particles, and 0·5 ml. injected into 21-day-old White Leghorn Chick No. 3324. A cell suspension was filtered through a Seitz clarifying pad and the filtrate injected into 25-day-old White Leghorn Chicks No. 3312, 3316, 3317, 3329. No tumours grew in any of these birds at the site of injection.

### EXPLANATION OF PLATE.

FIG. 1.—GRCH 16. Fowl 3086 original tumour. Spindle cell sarcoma induced with methylcholanthrene. H. & E. × 900.

FIG. 2.—GRCH 17. Fowl 3085 original tumour. Spindle cell sarcoma infiltrating between and invading voluntary muscle fibres which are seen in cross section. Note strap-like interlacing character of tumour cells. H. & E. × 900.

FIG. 3.—Petri dish culture of ascending paper chromatograms. Reading from left to right: GRCH 16 + *B. prodigious*, MH2 + *B. prodigiosus*, sterile control, *B. prodigiosus* control. Incubated 48 hours at 37° C. and allowed to colour on bench for 24 hours.

FIG. 4.—Giant cell and fibroblast reaction tissue around sterile No. 1 Whatman paper pulp injected 198 days previously. Well formed collagen appears black. Van Gieson. × 480.

FIG. 5.—Fowl 3309. Rous Sarcoma No. 1 at site of injection in left pectoral muscle region and localisation in left wing web by nitric acid cauterisation 8 days later. The bird was killed 14 days after cauterisation. The skin just above the pectoral tumour was torn accidentally *post mortem.*

FIG. 6.—Fowl 3328. Rous Sarcoma No. 1 injected in right pectoral muscles with reconstituted tumour. Cauterised 6 days later in right wing. Section shows localisation of very early typical Rous sarcoma at site of cautery. H. & E. × 45.

FIG. 7.—Fowl 3896. Injected intraperitoneally with MH2 homogenate. Killed 24 days later with extensive intraperitoneal tumour spread and localised tumour around wing tab. × 1½.

FIG. 8.—Fowl 3896. MH2 endothelioma at site of inflammatory focus around wing tab. A tendency to syncytial growth is characteristic of this tumour. × 170.

FIG. 9.—Fowl 3285. GRCH 17/2 in Rous-immune bird. H. & E. × 1000.

1
2
3
4
5

6

7

8

9

23.vi.50 : No. 3324, showing an early palpable tumour at the site of injection in the pectoral muscles, was cauterised in the right wing with a single thermo-cautery puncture, and in the left wing with a single application of a glass rod moistened with fuming nitric acid.

7.ix.50 : No. 3324 was killed. There was a large tumour at the site of injection, but no tumour at either the site of the thermo-cautery or the acid burn, both of which had completely healed within 10 days.

Histologically the tumour was typical GRCH 17 sarcoma. Fresh cell suspension of this tumour was injected into Fowls No. 3312, 3316 and 3317, which had not grown tumours at the site of injection of the Seitz filtrate (see above). Typical GRCH 17 sarcoma grew in No. 3312 and 3316 at the site of injection of cell suspension.

Fresh cell suspension from the tumour in No. 3316 was injected into the left breast of No. 3317, which again failed to grow a tumour.

Thus the GRCH 17 tumour grew in a Rous-immune bird and did not acquire the Rous agent, as far as could be demonstrated by tests in further serial passage.

### DISCUSSION.

The undoubted role of viruses in the experimental propagation of a number of malignant tumours in fowls justifies prolonged search for similar viruses in other spontaneous or induced tumours. Over a period of 20 years we have carried out such research for viruses in chemically-induced tumours without obtaining either direct or indirect evidence pointing to such factors as being involved in their aetiology. Nevertheless, it is difficult to exclude the possibility. The peculiar type of localisation associated with proliferative reactions in birds bearing virus-induced tumours has been less easily demonstrable with MH2 endothelioma than with the other virus tumours in our experience. On the other hand, no such localisation has been observed in the case of the GRCH series of tumours.

It seemed that the electron microscope, which can undoubtedly reveal particles of the size of viruses, might afford conclusive evidence of their presence or absence in tumour cells. In our limited experience of this technique, however, there are even less differences to be seen between infective and non-infective tumours and even normal tissues and in homogenates and filtrates prepared from them than can be seen by ordinary visual microscopy or even by direct visual observation. Further work may overcome this initial difficulty of recognising viruses from normal cell constituents ; but in the meantime circumstantial evidence points to essential differences between certain induced and spontaneous tumours.

The occasional occurrence of a chemically-induced tumour which on propagation yields a virus does not in our opinion justify the assumption that all tumours contain such viruses. Indeed, the existence of spontaneous filterable tumours would suggest that ultimately one is likely to encounter such agents in birds injected with carcinogens. Duran-Reynals (1952) has recently suggested, however, that viruses not usually associated with tumours may become adapted to cells conditioned by chemical carcinogens, and so play a part in the aetiology of cancer. By painting with methylcholanthrene the skin of birds carrying fowl pox virus he induced histologically malignant epithelial tumours, the cells of which contain characteristic inclusions. Filtrates of such tumours, however, reproduce fowl pox, not cancer. Our failure to induce any epidermoid cancers in the skin of fowls after repeated painting with chemical carcinogens may be due, as he suggests, to the absence of fowl pox in our flock; but we have had no difficulty in inducing upwards of 60 sarcomata at the sites of injection of chemical carcinogens, and have seen a few carcinomata of the crop following exposure to 2-acetylaminofluorene (Peacock and Peacock, 1949). Though we failed in attempts to transmit the carcinomata and therefore cannot assess their possible viral content, we saw no inclusions of the kind described and illustrated by Duran-Reynals (1952) in his fowl pox methylcholanthrene carcinomata.

The study of a wider range of chemically-induced fowl tumours, preferably including carcinomata of recognisable patterns, seems to offer the best prospects for obtaining more information about the aetiological factors concerned. At the present stage of our knowledge it seems unwise to generalise about viruses in relation to all tumours.

### SUMMARY.

1. Three new transplantable, chemically-induced fowl sarcomata, GRCH 16, 17, 18, are described.

2. Mill Hill 2 endothelioma has been successfully transmitted by desiccate kept at 0° C. for 12 years.

3. Comparisons between Rous Sarcoma No. 1, Mill Hill 2 endothelioma and the GRCH 16, 17, 18 sarcomata show that the two former tumours are readily transmissible by filtrates and by other extracts in which cells are probably destroyed or excluded, whereas the GRCH tumours are only transmissible by extracts containing cells.

At the time of writing the authors were unaware that Loewenthal (1931) had used capillary ascent on strips of filter paper of extracts of Rous Sarcoma No. 1 as a means of separating the virus from cells, and achieved the same results as we subsequently obtained.

This work forms part of a programme of virus research supported by the British Empire Cancer Campaign.

### REFERENCES.

Baker, S. L., and Peacock, P. R.—(1926) *Brit. J. exp. Path.*, **7**, 310.
Bedson, S. P., and Bland, J. O. W.—(1929) *Ibid.*, **10**, 67.
Begg, A. M.—(1927) *Lancet*, i, 912.
Duran-Reynals, F.—(1952) *Ann. N. Y. Acad. Sci.*, **54**, 977.
Loewenthal, H.—(1931) *Z. Krebsforsch.*, **34**, 551.
Peacock, P. R.—(1946) *Cancer Res.*, **6**, 311.
Peacock, A., and Peacock, P. R.—(1949) *Brit. J. Cancer*, **3**, 289.
Pentimalli, F.—(1916) *Lo Sperimentale*, Anno LXX, fasc. 3–4.
Rous, P., Murphy, J. B., and Tytler, W. H.—(1912) *J. Amer. med. Ass.*, **58**, 1751.
Rous, P., and Smith, W. E.—(1945) *J. exp. Med.*, **81**, 597.